# Supplementary material for: Phylogeography and Biological Characterizations of H12 Influenza A Viruses
Source: Viruses. 2022 Oct 13;14(10):2251. doi: 10.3390/v14102251 (PMC9610781; doi:10.3390/v14102251)
Supplement: Supplementary file 1 [file viruses-14-02251-s001.zip › viruses-1931083-supplementary.pdf]

Table S1 Statistically supported host transition of H12 IAVs estimated from HA gene

From	To	Bayes factor[a]	Posterior probability[b]
Anas	*Arenaria*	155659.271	1
Anas	*Uria*	155659.271	1
Arenaria	*Cygnus*	155659.271	1
Arenaria	*Mareca*	31118.018	0.999
Anas	*Calidris*	19442.275	0.999
Arenaria	*Leucophaeus*	10361.142	0.998
Larus	*Anas*	645.158	0.973
Arenaria	*Sibirionetta*	410.387	0.960
Environment	*Somateria*	362.403	0.954
Larus	*Shorebird*	308.388	0.947
Larus	*Environment*	64.382	0.788
Larus	*Leucophaeus*	41.163	0.704
Anas	*Guineafowl*	40.023	0.698
Cygnus	*Larus*	28.291	0.621
Anas	*Bucephala*	23.704	0.578

[a] Statistically supported transitions with Bayes factor>3 are presented,

[b] Posterior probability > 0.5 suggests well support viral transmission.

Table S2 Statistically supported migration rates of H12 IAVs estimated from HA gene

From	To	Bayes factor[a]	Posterior probability[b]
**Asia**	Oceania	29353.79	1
**Europe**	South America	4889.581	0.999
**Europe**	Oceania	47.267	0.935
**Europe**	Asia	38.321	0.922
**North America**	Europe	31.399	0.901
**North America**	Asia	10.355	0.760

[a] Statistically supported transitions with Bayes factor >3 are presented,

[b] Posterior probability > 0.5 suggests well support viral transmission.
